# Conium and Opium

**Published:** 1842-01

**Authors:** 


*Conium and Opium.*—The following comparative view of the respective effects of conium and opium, is from a paper in the Med. Bot. Trans., vol. 1. pt. 4, by Mr. Judd:

| Conium. | Opium. |
|---|---|
| Brain unnaturally free from blood; ventricles almost dry. | Brain gorged with blood; ventricles full of serum. |
| Lungs empty of blood. | Lungs so full of blood, that it runs out in a stream on their being cut. |
| Villous coat of the stomach and esophagus, white. | Villous coat and esophagus red. |
| Right side of the heart gorged with blood; left side empty. | The heart's cavities all contain blood. |
| Heart dead to stimuli. | Unknown whether so or not. |
| Intestines active and white. | Intestines inactive, red and inflamed. |
| Blood dark and fluid in both veins and arteries. | Blood at times, but not always, fluid. |
| Death of the heart. | Death, as I conclude from the other symptoms, of the brain. |

From his comparative experiments with the two drugs, Mr. J. doubts whether conium ever produces sleep, as opium does, by rendering the vessels of the brain turgid. From the power displayed in his experiments by conium, in lessening the quantity of blood sent to the brain and spinal cord, and also in lessening the force of the heart's action, Mr. J. concludes that it may be a valuable remedy in hypertrophy of the heart, in phrenitis and in inflammation of the spinal marrow. The experiment is worth trying.—*N. Y. Med. Gaz.*

